# The Blood-Brain Barrier and Microvascular Water Exchange in Alzheimer's Disease

**DOI:** 10.1155/2011/615829

**Published:** 2011-05-04

**Authors:** Valerie C. Anderson, David P. Lenar, Joseph F. Quinn, William D. Rooney

**Affiliations:** ^1^Department of Neurological Surgery, Oregon Health & Science University, 3181 SW Sam Jackson Park Road, Portland, OR 97239, USA; ^2^Department of Neurology, Oregon Health & Science University, Portland, OR 97239, USA; ^3^Advanced Imaging Research Center, Oregon Health & Science University, Portland, OR 97239, USA

## Abstract

Alzheimer's disease (AD) is the most common form of dementia in the elderly. Although traditionally considered a disease of neurofibrillary tangles and amyloid plaques, structural and functional changes in the microvessels may contribute directly to the pathogenesis of the disease. Since vascular dysfunction often precedes cognitive impairment, understanding the role of the blood-brain barrier (BBB) in AD may be key to rational treatment of the disease. We propose that water regulation, a critical function of the BBB, is disturbed in AD and results in abnormal permeability and rates of water exchange across the vessel walls. In this paper, we describe some of the pathological events that may disturb microvascular water exchange in AD and examine the potential of a relatively new imaging technique, dynamic contrast-enhanced MRI, to quantify water exchange on a cellular level and thus serve as a probe of BBB integrity in AD.

## 1. Introduction

Alzheimer's disease (AD) is the most common form of irreversible dementia in the elderly and accounts for more than 30% of all cases in adults over the age of 80 [1]. Pathologically, the disease is characterized by amyloid deposits, neurofibrillary tangles, and neuronal loss in specific brain regions. Vascular involvement is not part of the diagnostic criteria. Nevertheless, factors that modify vascular risk, including hypertension, diabetes, and hypercholesterolemia, are among the most consistently identified risk factors for the disease. Moreover, profound alterations in cerebrovascular ultrastructure and function have been identified in AD [2, 3]. Since the microvessels are the key site for nutrient and oxygen exchange between the brain and circulating blood, it is likely that processes that disturb capillary physiology or alter brain microcirculation are of major importance for the pathogenesis of AD [4–6].

**Figure 1:**
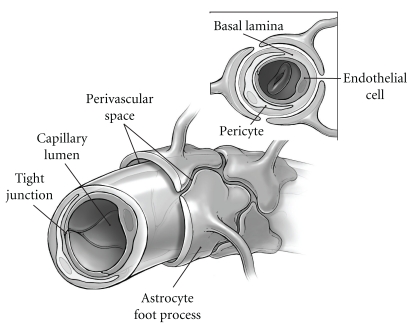
Schematic diagram of the Blood-Brain Barrier. Interstitial fluid flows along perivascular drainage pathways defined by the abluminal capillary surface and astrocyte end-feet, which ensheathe the vessels.

Morphologically, brain capillaries consist of a layer of endothelial cells that line the luminal surface, pericytes, and an outer basal membrane (Figure 1). In contrast to most of the peripheral endothelia, endothelial cells in the CNS form tight, unfenestrated junctions that restrict paracellular diffusion of water, ions, and large molecules. Since other mechanisms by which blood-borne substances cross into the brain (e.g., carrier-mediated active or facilitated transport, pinocytosis) are limited, these tight junctions limit the diffusion of blood-borne solutes into the brain and are the foundation of the Blood-Brain Barrier (BBB) [7]. In addition to the endothelium, the functional integrity of the barrier is also critically dependent on the basal lamina, pericytes, and surrounding astroglia. The basal lamina provides structural support for the endothelium, signals cell-cell interactions, and separates the endothelium and pericytes from the surrounding extracellular (interstitial) spaces. The pericytes, closely associated with the abluminal surface of the basal membrane, likely play a role in regulating microvascular blood flow and vascular remodeling [2]. Finally, the perivascular astrocyte end-feet which ensheathe the outer surface of the microvessels are of major importance in induction and maintenance of the tight junctions, neurovascular coupling, and fluid balance [8, 9]. 

Approximately 90% of the blood volume in the brain is water, and its exchange into and out of the blood is also tightly regulated by the BBB. As in the periphery, water in the CNS is highly compartmentalized and is present in all brain compartments: intracellular and interstitial fluids, blood, and CSF. Increasing evidence suggests that functional interactions between the cellular components of the BBB—the endothelium, basal lamina, and pericytes—in addition to the astroglial end-feet collectively regulate water exchange between compartments, capillary blood volume, and permeability. We hypothesize that microvascular water exchange is disturbed in the AD brain as a result of an incompetent BBB and is reflected in abnormal intercompartmental water exchange. In this paper, we will provide the rationale for our hypothesis and suggest a quantitative experimental approach, dynamic contrast-enhanced magnetic resonance imaging (DCE-MRI), by which we have recently begun to test this hypothesis in individuals with early cognitive changes.

## 2. The Pathophysiology of Water Regulation in Alzheimer's Disease

The morphological footprint of BBB disturbances in AD is clear. In the basal membrane, substantial thickening and stiffening is observed in 90% of AD cases [10]. Macroscopically, the capillaries appear thin and fragmented [11]. Overall density tends to be reduced, especially in important cortical and hippocampal regions, and remaining vessels are more tortuous. In addition, the endothelium is frequently atrophic or swollen, and physical coupling to the surrounding glia is often disrupted [12]. Moreover, the number of tight junctions per unit of vessel length is reduced throughout the brain [13–15] with metabolic regulation of remaining tight junctions likely compromised by decreased, mitochondrial density [14]. On a molecular level, the cell adhesion activity of occludin and claudins, integral member proteins localized exclusively to tight junctions, is decreased and accumulation of collagen deposits, proteoglycans, laminins, and other components of the basal matrix is frequently noted [16–18].

Despite the overwhelming abnormalities in capillary structure and function, studies to define the temporal association of microvascular changes with disease severity and progression have not been done in humans, and the extent to which BBB changes are likely to be symptomatic of or causal to the disease remains unclear. Contributing to the uncertainty is the variety of pathological environments present in the AD brain. We expect that BBB function is most likely disturbed as the result of multiple pathologic processes, each of which may influence water exchange at the BBB, as discussed briefly in the following. 

### 2.1. Cerebral Amyloid Angiopathy (CAA)

Amyloid-*β* peptides (A*β*) are derived from proteolytic cleavage of the transmembrane amyloid precursor protein (APP) and vary in length from 39–43 amino acids [19]. In AD, deposits of A*β* characteristically accumulate in the parenchyma as plaques. However, deposition of insoluble A*β* in the vessel walls and interstitial spaces (as cerebral amyloid angiopathy (CAA)) occurs in nearly all individuals with AD [20]. Vascular A*β* deposits are not always found in association with AD, but, when they are found, they exhibit several important differences from non-AD CAA [21]. In particular, the deposits are most commonly associated with the capillaries, where they attach to basal lamina and frequently occlude the lumen and/or protrude into the interstitial space [22–24]. In addition, the deposits are enriched in A*β*_1–42_, the specific isoforms of A*β* found in neuritic plaques [25]. 

Increasing evidence suggests that these deposits may affect BBB function in AD [26, 27]. In culture, exposure of cortical microvessels to A*β*_1–42_ directly damages the endothelium, resulting in an abnormal plasma membrane pattern, reduced expression of tight junction protein complexes, and increased permeability [28]. *In vivo*, there is clear evidence that vascular A*β*_1–42_ deposits are associated with microhemorrhages. In the human AD brain, both microhemorrhages and A*β*_1–42_ deposits are found close to or encircling microvessels, show densities that covary throughout the brain, and contain both blood- and vessel-derived proteins (fibrinogen, von Willebrand factor, collagen VI) [29]. Moreover, recent studies in APP transgenic mice have shown that increased vascular A*β*_1–42_ levels are associated with decreased capillary density and abnormal basement membrane protein composition, providing evidence that A*β*_1–42_ accumulation is sufficiently destructive to cause loss of vessels *in vivo* [30]. Finally, on a macroscopic scale, CAA-associated A*β*_1–42_ deposits essentially recapitulate the perivascular drainage pathways [31, 32]. Thus, A*β*_1–42_ deposits build up around the same abluminal surfaces along which interstitial fluid is cleared, impeding diffusion of fluids and further compromising the BBB's ability to regulate water effectively.

### 2.2. Inflammation

Blood-Brain Barrier function may also be affected by the inflammatory environment of AD microvessels [33]. Endothelial cells and astrocytes are activated during inflammatory CNS disease and express a variety of angiogenic mediators that affect BBB permeability, as demonstrated in other experimental models. For example, in experimental allergic encephalopathy, vascular endothelial growth factor (VEGF-A) localized in reactive astrocytes is upregulated and decreases expression of tight junction proteins, converting the microvessels into permeable fenestrated capillaries [34]. Importantly, affected vessels are no more permeable to proteins and macromolecules than those with tight junctions but are much more permeable to water. 

In AD, many microvessels express the same growth factors, proteases, and proteins that typically characterize an angiogenic response. However, the extent to which expression of these factors is related to angiogenesis is unclear [35–37]. APP transgenic mice overexpressing A*β* exhibit impaired angiogenesis [38]. In the human AD brain, both microvascular density [11, 39] and reduced blood volume [40–42] are commonly observed. It is possible that the growth factors and other markers typical of an angiogenic response may in fact mediate an inflammatory one in the context of the increased A*β* levels that characterize the AD brain, with direct effects on the BBB. Consistent with this, A*β* has been shown to stimulate expression and activation of metalloproteases that degrade a wide variety of extracellular matrix components, resulting in loss of tight junctions and BBB integrity [36, 43].

### 2.3. Aquaporins

In addition to inflammatory mediators, astrocytes in the brain express aquaporin-4 (AQP4), one of the family of water channels found in plasma membranes throughout the body [44–48]. Functionally, aquaporins regulate transmembrane water permeability in response to osmotic gradients. In the brain, AQP4 is localized to tissue-fluid interfaces: in the glia limitans (pia-subarachnoid CSF), the ependyma (ependymal lining-ventricular CSF), and at the BBB in the astrocyte foot processes and, to a lesser extent, the endothelium [48]. The expression of AQP4 specifically at the borders of fluid-filled compartments suggests an important role of these channels in water homeostasis, a role now confirmed by many groups [45, 49–52].

While evidence linking aquaporins with fluid regulation in conditions associated with brain edema is now substantial, the effect of neurodegenerative disease on aquaporins and the consequences to BBB function remain to be defined [44]. At present, the extent to which AQP4 pathophysiology contributes to structural abnormalities in the BBB has not been established [53, 54]. Nevertheless, Wilcock et al. have recently found that AQP4 localization to the perivascular end-feet is significantly reduced in APP transgenic mice with high vascular A*β*, as is the density of astrocyte end-feet in close contact with vessel wall [55]. That only minimal changes were observed in APP transgenics with low A*β* load suggests that aquaporin function may be altered at the BBB in AD and may be a consequence of A*β* deposition in the microvessels.

## 3. Dynamic Contrast-Enhanced Magnetic Resonance Imaging (DCE-MRI)

The experimental index of BBB integrity has traditionally been based on the exclusion of blood-borne molecules (e.g., albumin or horseradish peroxidase) for which BBB transport mechanisms are poor. In the case of albumin, a 70 kDa serum protein, the albumin transporter is nearly absent in brain endothelium, while horseradish peroxidase is rarely found in the parenchyma due to the absence of pinocytotic vesicles [4, 56]. The presence of low molecular weight dyes in the cerebrospinal fluid (CSF) after intravenous injection has also been used to probe BBB compromise. While methodologically simpler, these experiments can be difficult to interpret as details related to dye stability, binding mechanisms, and specific effects on vascular morphology are generally lacking. Nevertheless, an age-related increase in Evans blue and carboxyfluorescein has been observed in the cortex of APP transgenic mice overexpressing A*β* following rapid intraperitoneal injection [57]. Importantly, changes in permeability appeared in young mice, *before* A*β* deposition, consistent with BBB changes early in the disease process. However, findings have not been universal and in double transgenics overexpressing APP and presenilin 1, part of the *γ*-secretase complex responsible for APP cleavage, bolus infusion of neither albumin nor ^125^I-insulin showed increased permeability compared to age-matched controls [58]. 

Assessment of BBB function in AD patients has been limited for the most part to analysis of cerebrospinal fluid (CSF) content. Here, too, data are conflicting, and albumin levels significantly different from those of age-matched controls have not been consistently identified [59, 60]. Nevertheless, an increased CSF-albumin index has been reported in subsets of AD patients by several groups [60]. Additionally, Bowman et al. recently found a significant correlation of CSF-albumin index with the rate of disease progression in a subset of patients with mild-to-moderate AD [61]. This finding suggests that BBB dysfunction may increase the rate of disease progression in at least some AD patients. 

In contrast to this more traditional approach, dynamic MRI techniques provide quantitative measures of BBB integrity based on changes in the water proton (^1^H_2_O) longitudinal or transverse relaxation rate constants, *R*_1_ and *R*_2_*, respectively, during bolus passage of a low molecular weight paramagnetic contrast reagent (CR). Dynamic susceptibility contrast (DSC) MRI, is based on measurement of *R*_2_*  ( = 1/*T*_2_*) effects and has been used by many groups to characterize perfusion changes in the AD brain [62–65]. *R*_2_* effects can be exquisitely sensitive to pathophysiological changes, but their interpretation on a molecular level can be challenging. Changes in *R*_2_* are strongly influenced by bulk magnetic susceptibility effects. These effects are long range and vary depending on the size, shape, and orientation of the local magnetic field [66]. As a result, susceptibility effects not only cross tissue compartment boundaries but vary substantially on the histological scale, which is small with respect to an MRI voxel [67–69]. Susceptibility effects, therefore, can make analytical interpretation of DSC-MRI problematic and result in large errors in pharmacokinetic estimates derived from DSC measurements [70]. In contrast, DCE-MRI is based on *R*_1_  ( = 1/*T*_1_) changes. These occur from direct contact of ^1^H_2_O to CR and are not influenced by bulk magnetic susceptibility [71]. As a result, the ^1^H_2_O  *R*_1_ value in a given compartment is unaffected by CR in an adjacent compartment except by intercompartment ^1^H_2_O exchange. Thus, *R*_1_ changes during bolus CR passage can be interpreted analytically to provide a quantitative measure of intercompartmental water dynamics. Changes in tissue water compartmentalization have been shown to be an extremely sensitive and early indicator of BBB breakdown in multiple sclerosis, tumors, and other pathologies [72–74].

**Figure 2:**
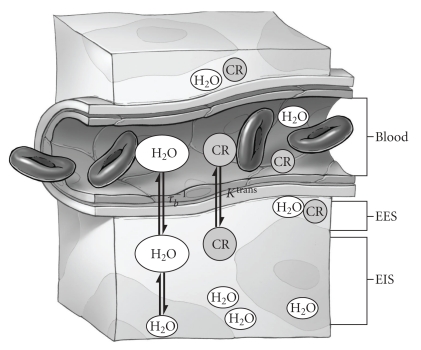
Schematic diagram of the three-compartment model of tissue. EES: extravascular extracellular space; EIS: extravascular intracellular space; *τ*_*b*_^−1^: unidirectional rate constant for water extravasation; *K* ^trans^: pseudo-first-order rate constant for CR extravasation.

 The accuracy of DCE-MRI parameters depends critically on the pharmacokinetic model used to fit the tissue *R*_1_ changes (Δ*R*_1*t*_) after CR injection. To a first approximation, biological tissue can be described by three compartments: blood, extravascular extracellular (EES), and extravascular intracellular space (EIS) (Figure 2). In each compartment, CR and ^1^H_2_O are assumed to be well mixed. In healthy brain, ^1^H_2_O (which forms the basis for the MR signal) occupies and exchanges between all three compartments, while low molecular weight contrast reagents do not permeate cell membranes and are restricted to the plasma and EES [67–69]. Immediately after injection, CR is confined to the plasma and greatly increases the *R*_1_ of ^1^H_2_O in the blood, *R*_1*b*_(^1^H_2_O exchange between erythrocytes and plasma is fast on the MR timescale, and the amount of ^1^H_2_O in blood can be modeled using the hematocrit volume fraction) [75]. Over time, CR diffuses through the vessel wall and increases the *R*_1_ of ^1^H_2_O in the extravascular space. Thus, the mathematical relationship between *R*_1*b*_ and *R*_1*t*_ depends not only on the kinetics of compartmental ^1^H_2_O exchange but also on the rate at which CR leaks through the vessel wall (*K* ^trans^) [76].

In the limit of small *K* ^trans^  (<10^−4^ min ^−1^), as is the case for studies of normal and near-normal BBB permeability, a model with only two compartments is sufficient to describe transendothelial ^1^H_2_O exchange. In this two-site model, it is assumed that CR is initially confined to the blood plasma and that ^1^H_2_O freely exchanges between the plasma and a combined (EES and EIS) extravascular space. Since most of the ^1^H_2_O MRI signal originates from the extravascular space (in white matter, the blood ^1^H_2_O signal is less than 2% of the total signal), it is further assumed that *R*_1*t*_ exhibits single exponential behavior. At early times after CR administration, the time dependence of *R*_1*t*_ changes depend primarily on changes in *R*_1*b*_, and hence on the concentration of CR in the blood (CR_*b*_); [CR_*b*_] is a fictious concentration since CR distributes only into the plasma, so it is useful to recast this in terms of [CR_*p*_]:

(1) $$\begin{array}{cc} R_{1b}\left(t\right) & =r_{1}\left[{\text{CR}}_{b}\right]\left(t\right)+R_{1b0}=r_{1}\;\left(1-\text{h}\right)\left[{\text{CR}}_{p}\right]\left(t\right)\;+\;R_{1b0}, \end{array}$$

where *r*_1_ is the longitudinal relaxivity of CR, *R*_1*b*0_ is the *R*_1_ of blood ^1^H_2_O before CR injection and h is the hematocrit.

CR extravasation also contributes to the time dependence of Δ*R*_1*t*_, and this is accounted for by a time-varying extravascular *R*_1_  (≡*R*_1*e*_) component. As CR permeates the BBB, it passes into the EES (see Figure 2). If *K* ^trans^ is small, though, CR never achieves sufficient concentration to drive the EIS-EES water exchange. Under these conditions, the linear relationship of (2) applies. Here, [CR_EES_] is the concentration of CR in the EES. Under these conditions, the time dependence of [CR_EES_], and hence *R*_1*e*_, is determined by the Kety-Schmidt integral rate law [72, 79, 80]. Manipulation of these two equations yields the (nonlinear) relationship between *R*_1*b*_ and *R*_1*t*_ for two-site transendothelial exchange shown in (3) [67–69]. Fits of *R*_1*b*_ and *R*_1*t*_ to (3) yield not only *v*_*b*_, the cerebral blood volume (*v*_*b*_ = *p*_*b*_ *f*_*w*_, where *f*_*w*_ is the tissue volume accessible to mobile solutes (ca. 0.8)), but *τ*_*b*_^−1^, the rate constant for water extravasation. *τ*_*b*_^−1^ and the related permeability-surface area product of water, *P*_*w*_ *S*  ( = *v*_*b*_/*τ*_*b*_), represent quantitative measures of capillary water permeability and are direct measures of BBB integrity. Here we assume that *r*1 is independent of compartment:

(2) $$\begin{array}{c} R_{1e}\left(t\right)=r_{1}v_{e}\left[{\text{CR}}_{\text{EES}}\right]\left(t\right)+R_{1e0}, \end{array}$$

where *v*_*e*_ is the extravascular extracellular volume fraction and *R*_1*e*0_ is the *R*_1_ of the extravascular ^1^H_2_O before CR injection and without transendothelial exchange,

(3) $$\begin{array}{cc} R_{1t}\left(t\right) & =\frac{1}{2}\{\left[R_{1b}\left(t\right)+R_{1e}+\tau_{b}^{-1}+\frac{p_{b}}{\tau_{b}\left(1-p_{b}\right)}\right]\\ & \;\;\;-[{\left(R_{1e}-R_{1b}\left(t\right)-\tau_{b}^{-1}+\frac{p_{b}}{\tau_{b}\left(1-p_{b}\right)}\right)}^{2}\\ & \;\;\;\;\;{\;+\frac{4p_{b}}{\tau_{b}^{2}\left(1-p_{b}\right)}]}^{1/2}\}, \end{array}$$

where *R*_1*t*0_, *R*_1*b*0_ are the *R*_1_ of tissue and blood, respectively, before CR injection, *R*_1*e*_ is the *R*_1_ of extravascular water in the absence of transendothelial exchange, *τ*_*b*_ is the average intravascular lifetime, and *p*_*b*_ is the mole fraction of blood water.

**Figure 3:**
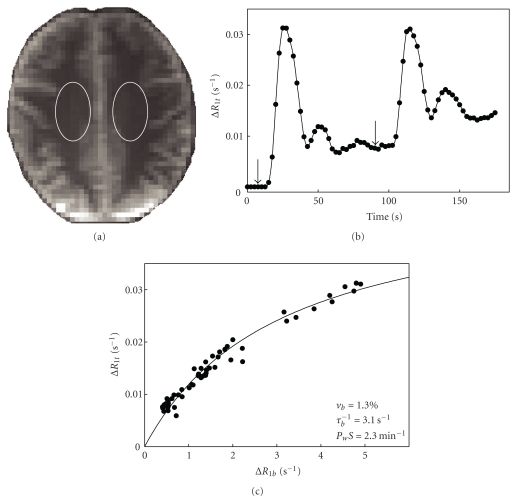
DCE-MRI region of interest (ROI) analysis in the centrum semiovale (CSO) of a 73-year-old healthy female. (a) Axial *T*_1_-weighted (turboFLASH) images were collected on a Siemens 7 T system with 8-channel phased array transmit/receive head coil; selected ROI shown in white. (b) Plot of pre-post contrast *R*_1*t*_ (Δ*R*_1*t*_) versus time. Images were collected immediately preceding two 0.05 mmol/kg bolus injections of gadoteridol and every 2.5 seconds over the next 175 sec. *R*_1*t*_ values were calculated at each time point by fitting the signal intensity curves to a standard two-parameter single exponential inversion recovery equation [77]. (c) Δ*R*_1*t*_ plotted as a function of Δ*R*_1*b*_. Changes in the blood signal, Δ*R*_1*b*_, were determined from an ROI placed entirely in the sagittal sinus. The solid line represents the best fit of the data to (3). Resultant estimates of *v*_*b*_, *τ*_*b*_^−1^, and *P*_*w*_ *S* are also shown.

**Figure 4:**
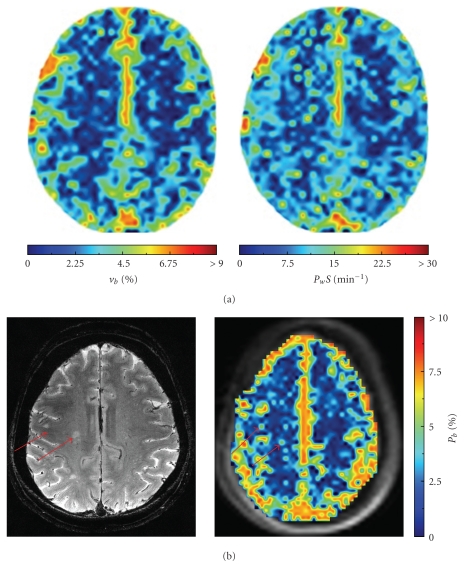
7 T (a) DCE-MRI maps of blood volume (*v*_*b*_) and water permeability (*P*_*w*_ *S*) in a superior slice from a 71-year-old female with early AD [78]. (b) *T*_2_-weighted spin echo image and corresponding *p*_*b*_ map from a healthy 70-year-old female. Arrows indicate hyperintense white matter regions (15–23 mm^2^) visible on both the *T*_2_ image and corresponding parametric map.

Previous DCE-MRI studies in AD individuals have found minimal disruption of the BBB [81, 82]. However, these studies are limited by the relatively low field strength (1.5 T) of the measurements and the lack of pharmacokinetic modeling. The real power of DCE to probe BBB disturbances, particularly in the context of a relatively intact barrier, is most evident at high field, where the increased signal-to-noise and reduced CR detection threshold yields significantly better precision and accuracy of pharmacokinetic estimates.  Figure 3 shows a representative 7 T DCE-MRI study performed recently in our laboratory. Fitting to (3) yields values of *τ*_*b*_^−1^ and *v*_*b*_ in the centrum semiovale that are in close agreement with those reported previously [83, 84]. Application of (3) on a pixel-wise basis results in parametric maps like the ones shown in Figure 4(a) [78]. As far as we are aware, this is the first map of the water permeability surface area product (*P*_*w*_ *S*) produced using dynamic MR techniques in an individual with early AD, and underscores the power of DCE to visualize even subtle changes in BBB water permeability (Figure 4(b)).

It should be noted that use of (3) can lead to large errors in parametric estimates if ^1^H_2_O exchange across the BBB or extravascular cell membranes (i.e., between EES and EIS in Figure 2) is slow on the timescale of DCE measurements [85]. In either of these situations, abstraction of accurate parameters requires a three-compartment model and a more comprehensive pharmacokinetic treatment. Such a model has been developed by Li et al. and is currently being applied in our laboratory [76].

## 4. Conclusion

The BBB plays a critical role in water homeostasis in the brain. Although the mechanism remains to be determined, converging evidence suggests that AD pathophysiology may disturb the BBB and disrupt intercompartmental water exchange. DCE-MRI is an extremely powerful and sensitive probe of water dynamics in the living brain. Pharmacokinetic modeling provides quantitative estimates of blood volume, vascular permeability, and rates of transendothelial water exchange. We expect that DCE-MRI studies, particularly at high field, will play a key role in unlocking the contribution of BBB dysfunction to the pathophysiology of AD.
